# Involvement of per- and polyfluoroalkyl compounds in tumor development

**DOI:** 10.1007/s00204-024-03685-7

**Published:** 2024-03-13

**Authors:** Maija Pesonen, Kirsi Vähäkangas

**Affiliations:** Faculty of Health Sciences, School of Pharmacy/Toxicology, University of Eastern Finland, P.O. Box 1627, 70211 Kuopio, Finland

**Keywords:** PFAS exposure, Molecular mechanisms, Liver cancer, Kidney cancer, Testicular cancer, Breast cancer

## Abstract

Per- and polyfluoroalkyl substances (PFAS) are a large group of synthetic persistent chemicals, which are used in many industrial and commercial applications. Hundreds of different PFAS have been identified in the environment and they are commonly found also in human blood. Due to the chemical stability and extensive use, PFAS pose a risk for human health and wildlife. Mounting evidence indicates that PFAS-exposure adversely affects many organs including liver, kidney, and reproductive tissues and induces tumors in laboratory rodents. Epidemiological studies show association between PFAS-exposure and some tumors also in humans. Effects of PFAS-exposure are complex and obviously do not depend only on the concentration and the structure of PFAS, but also on age and sex of the exposed individuals. It has been difficult to show a causal link between PFAS-exposure and tumors. Moreover, molecular mechanisms of the PFAS effects in different tissues are poorly understood. PFAS are not directly mutagenic and they do not induce formation of DNA binding metabolites, and thus are assumed to act more through non-genotoxic mechanisms. In this review, we discuss the involvement of PFAS-compounds in tumor development in tissues where PFAS exposure has been associated with cancer in epidemiological and animal studies (liver, kidney, testicle and breast). We will focus on molecular pathways and mechanisms related to tumor formation following PFAS-exposure.

## Introduction

Per- and polyfluoroalkyl substances (PFAS) are a large group of manmade fluorinated chemicals, which are now present in the environment worldwide (Sunderland et al. 2019; Kurwadkar et al. 2022; Evich et al. 2022) and rather commonly detected in human blood (Calafat et al. 2007; Wielsøe et al. 2017; Li et al. 2020; Mancini et al. 2020). Increasing evidence indicates that PFAS-exposure can be involved in carcinogenesis by disrupting metabolic and immunologic pathways and inducing reproductive and developmental toxicity (Blake and Fenton 2020; Steenland et al. 2020; Fenton et al. 2021; Purdue et al. 2023). An increase of tumors in rodent tissues has been shown after dietary treatment with PFAS (Biegel et al. 2001; Butenhoff et al. 2012). In addition, epidemiological studies have reported an association between serum PFAS-levels and a risk of tumors in human tissues (Table 1). The increased risk of tumors has particularly been found in occupationally exposed individuals and in residents living in PFAS contaminated environment. Drinking water and contaminated air have often been identified as the source of PFAS exposure (Vieira et al. 2013; Mastrantonio et al. 2018; Bartell and Vieira 2021; Li et al. 2022a). The International Agency for Research on Cancer (IARC) has classified PFOA as a possible carcinogen in humans (class 2B, IARC 2016).

**Table 1:** Examples of PFAS-exposure in association with kidney, testicular, breast and liver cancers

References	Study design Participants (n)	PFAS	Cancer type	OR or HR^2^ (95%Cl)
Vieira et al. (2013)	Case–control 61 cases 7339 controls	PFOA	Kidney, testicle	2.0 (1.0, 3.9) 2.8 (0.8, 9.2)
Barry et al. (2013)	Community cohort 32,254 residents	PFOA	Kidney, testicle	1.10 (0.98, 1.24) 1.34 (1.00, 1.79)
Purdue et al. (2023)	Nested case–control 530 patients 530 controls	PFOS	Testicle	2.60 (1.1, 6.4)
Shearer et al. (2021)	Case–control 324 patients 324 controls	PFOA	Kidney	1.71 (1.23, 2.37)
Li et al. (2022a)	Cohort 60,507 participants	PFOA PFOS PFHxS	Kidney, testicle	1.27 (0.85, 1.89) 1.28 (0.73, 2.15)
Bonefeld-Jørgensen et al. (2011)	Case–control	PFOA	Breast	1.20 (0.77, 1.88)
Wielsøe et al. (2017)	Case–control 77 patients 84 controls	PFOA, PFHxS	Breast	1.26 (1.01, 1.58) 1.16 (1.02, 1.32)
Mancini et al. (2020)	Nested case–control 194 patients 194 controls	PFOA PFOS	Breast	7.73 ER- (1.46, 41.08) 3.44 PR- (1.30, 9.10) 2.22 ER + (1.05, 4.69) 2.47 PR + (1.07, 5.56)
Li et al. (2022a, b)	Case–control 373 patients 657 controls	PFOA PFDA	Breast	2.39 (1.88, 3.05) 2.00 (1.54, 2,58)
Feng et al. (2022)	Case-cohort 226 patient 990 control	PFOA, PFHpA	Breast	1.35 (1.03, 1.78) 1.20 (1.02, 1.48)
Tsai et al. (2020)	Case–control 120 patients 119 control	PFOS	Breast	2.34 (1.02, 5.38)
Velarde et al. (2022)	Case–control 75 patients 75 controls	PFDOA, PFHxA	Breast	9.26 (2.54, 45.10) 2.66 (0.95, 7.66)
Goodrich et al. (2022)	Nested case–control 50 patients 50 controls	PFOS	Liver	4.5 (1.20, 16.00)
Itoh et al. (2021)	Case–control 405 patients 405 controls	PFOA, IsoPFOA	Breast	< 1.0
Hurley et al. (2018)	Case–control	PFOA PFOS PFUnDA PFNA PFHxS	Breast	< 1.0
Bonefeld-Jørgensen et al. (2014)	Case–control 250 patients 233 controls	PFHxS PFOSA	Breast	< 1.0

The data published in BubMed between 2010–2023

*PFDA* perfluorodecanoic acid, *PFDOA* perfluorododecanoic acid, *PFHxA* perfluorohexanoic acid, *PFHxS* perfluorohexane sulfonic acid, *PFHpA* perfluoroheptanoic acid, *PFHpxS* perfluoroheptane sulfonic acid, *PFOA* perfluorooctanoid acid, *PFOS* perfluorooctane sulfate, *PFNA* perfluorononanoic acid, *PFOSA* perfluorooctanesulfonamide, *PFUnDA* perfluoroundecanoic acid, *OR* odds ratio, *HR* hazard ratio

**Fig. 1:**
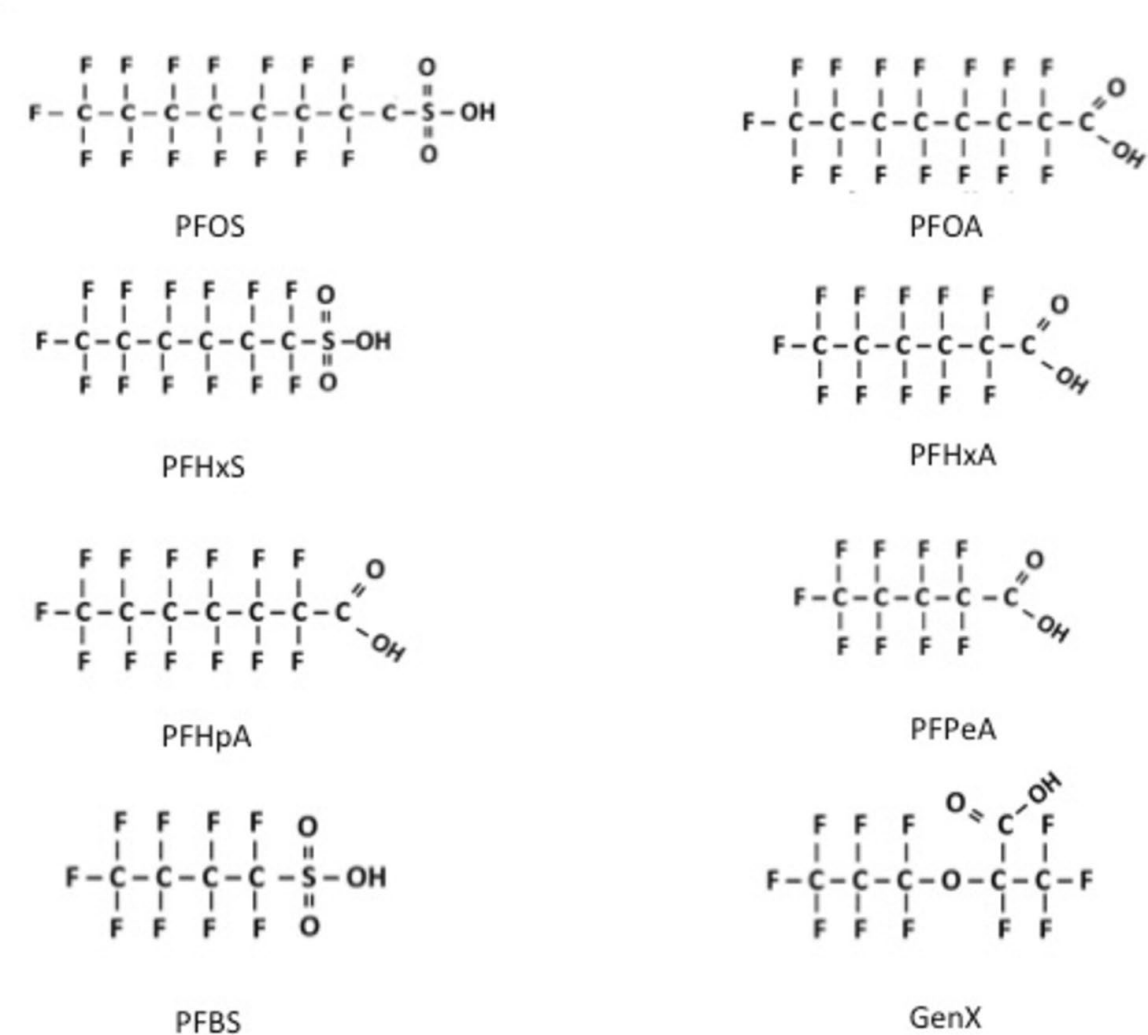
Some structures of PFAS that have been detected in biological samples

PFAS have been manufactured for about seven decades. They are used in many industrial and commercial applications such as firefighting foams, furniture, clothing, non-stick kitchenware, food packaging material, paints, and cosmetics (Sunderland et al. 2019; Evich et al. 2022). PFAS consist of a common aliphatic carbon backbone with different lengths and branches. The structure of several PFAS substances resembles endogenous fatty acids (Fig. 1). Research has generally focused on PFAS with four to sixteen carbons, and among these perflurooctanoic acid (PFOA) and perfluorooctane sulfate (PFOS) have been the most widely identified PFAS in humans and wildlife (Sunderland et al. 2019). PFAS are resistant to chemical, physical and biological transformation and accumulate in the food chain. Due to these properties the production and use of PFOA and PFOS have been restricted globally (EU 2020; UNEP 2009). Many alternatives to the restricted PFAS have been introduced on the global market. These PFAS are usually short-chain PFAS-compounds (< 6 carbon) such as perfluorobutane sulfonic acid (PFBS), perfluorobutanoic acid (PFBA), GenX or HFPO DA (ammonium salt of hexafluoropropylene oxide dimer acid; CAS: 62037-80-3) and some fluorotelomer alkohols (e.g., 4:2 ETOH, CAS: 2043-47-2 and 6:2 ETOH; CAS: 647-42-7) (Kjølholt et al. 2015; Solan et al. 2023). Although these substances have shorter half-lives, they do contain persistent carbon–fluorine (C–F) bonds and their toxicity and health effects are for the most part still unknown.

The main exposure-routes of humans and wildlife are ingestion of PFAS-contaminated food and water, or inhalation of contaminated air (Sunderland et al. 2019). PFAS have been demonstrated to cross placenta to a developing fetus, and newborn both in animals and humans can be exposed through breastfeeding (Blake and Fenton 2020; Varsi et al. 2022; Blomberg et al. 2023). The evaluation of human exposure to 17 PFAS-compounds in food samples obtained from 16 different European countries during 2007–2018 showed that the most prominent PFAS were PFOS, followed by PFOA, perfluorohexane sulfonic acid (PFHxS) and perfluorononanoic acid (PFNA) (EFSA 2018, 2020). Unlike other POP-compounds, PFAS do not primarily accumulate in the body fat but are distributed in plasma proteins (albumin, lipoproteins) mainly to the liver and kidney (Lau et al. 2007). Accumulation has also been reported in other tissues e.g., brain, lungs and reproductive tissues (Pérez et al. 2013).

In humans, PFAS are readily absorbed in the gastrointestinal tract and lungs but only slowly eliminated. There is no evidence that PFAS can be metabolized before excretion in urine and bile. In general, the elimination is enhanced as the carbon chain length decreases. There are large gender and species differences in the half-lives of PFAS; they are faster eliminated in females than in males, and faster in laboratory animals than in humans (Table 2). PFAS with known half-lives bind extensively to proteins, also to organic anion transporter proteins (OATs), and are reabsorbed in the proximal tubule of the kidney (Lau et al. 2007; Louisse et al. 2023). The variation in elimination between animals and humans complicates the use of animal data in human risk assessment (Lau et al. 2007; Olsen et al. 2007; Fenton et al. 2021).

**Table 2:** Some common PFAS substances and the estimated serum half-lives in three species (Olsen et al. 2007; Fenton et al. 2021)

Chemical name	Abbreviation	Formula	Human	Monkey	Rat
Perflurobutanoic acid	PFBA	C_4_HF_7_O_2_	3 d	1.7 d	1–9 h
Perfluorohexanoic acid	PFHxA	C_6_HF_11_O_2_	32 d	2.4–5.3 h	0.4–0.6 h
Ammonium salt of hexafluoropylene oxide dimer acid	GenX HFPO DA	C_6_HF_11_O_3_	4–6 d	NA	3–8 h
Perfluoroheptanoic acid	PFHpA	C_7_HF_13_O_2_	1.2–2.5	NA	1.2 -2.4 h
Perfluorooctanoic acid	PFOA	C_8_HF_15_O_2_	2.1–3.8 y	21–30 d	2–6 d
Perfluorononanoic acid	PFNA	C_9_HF_17_O_2_	2.5–4.3 y	NA	1.4–55 d
Perfluorodecanoic acid	PFDA	C_10_HF_19_O_2_	3.1–4.4 y	NA	NA
Perfluorobutane sulfonic acid	PFBS	C_4_HF_9_O_3_S	28 d	3.5–4.0 d	2.1–4.5 h
Perfluorohexane sulfonic acid	PFHxS	C_6_HF_13_O_3_S	5.3–8.5 y	87–141 d	1.8–6.8 d
Perfluorooctane sulfonic acid	PFOS	C_8_H_2_F_17_NO_2_H	3.4–5.0 y	110–132 d	62–41 d

*d* days, *h* hour, *y* year, *NA* not available

PFAS have been associated with adverse health effects in humans including the elevated serum cholesterol and liver enzyme levels, immunosuppression and pregnancy complications (DeWitt et al. 2019; Blake and Fenton 2020; Steenland et al. 2020; Fenton et al. 2021). Epidemiological studies have further reported associations between serum PFAS-levels and a risk of tumors in many organs including kidney, bladder, testis, prostate, breast, ovarian, liver and immune tissue (non-Hodgkin lymphoma) (Vieira et al. 2013; Mastrantonio et al. 2018; Bartell and Vieira 2021; Boyd et al. 2022; Goodrich et al. 2022; Li et al. 2022a; Purdue et al. 2023). The risk has most often been described following exposures to long-chain PFAS (e.g., PFOA, PFOS, PFHxA, PFDA) whereas the risk after exposure to short-chain PFAS is less studied. In rat, PFAS have been shown to induce tumors in several organs including liver, testicle and pancreas (Biegel et al. 2001; Lau et al. 2007; Butenhoff et al. 2012). There is no reported evidence of renal tumors after PFAS-treatment in rodents. However, there is epidemiological evidence of PFAS-related tumors in kidney, whereas no association between pancreatic tumors and PFAS-exposure in humans.

Although exposure to PFAS is known to have adverse health effects and potentially inducing tumors in many human tissues, molecular mechanisms of such effects are poorly understood. In general, PFAS have not been shown to be directly mutagenic or induce formation of DNA binding metabolites but assumed to act more through non-genotoxic mechanisms. These substances are not acutely toxic but act more chronically altering gene expression and reprogramming various molecular pathways. In this review, we will discuss molecular changes in PFAS-associated cancers in liver, kidney, testicular and breast tissues. These tissues were chosen because the liver and kidney are the main target organs where PFAS accumulate and PFAS have previously been shown to induce tumors in rodent liver and testicle. Furthermore, there is rather good epidemiological support for the risk of tumors in testicle, kidney and breast following PFAS-expose (Mancini et al. 2020; Bartell and Vieira 2021; Li et al. 2022a; Purdue et al. 2023). We will focus on molecular mechanisms and pathways related to tumors after PFAS-exposures in humans and laboratory animals.

## Tissue-specific mechanisms related to tumors

### Effects of PFAS in the liver

The liver is the main target organ of toxicity and accumulation of PFAS in humans and rodents. PFAS-levels (e.g., PFOA, PFOS, PFNA) in human blood have been associated with increased serum enzyme protein levels (AST, ALT, GGT), which generally indicate liver injury (Darrow et al. 2016; Costello et al. 2022). There is also evidence that PFAS-exposure dysregulates bile acid metabolism and contributes to the kind of lipid accumulation that is present in patients with non-alcoholic fatty liver (Jin et al. 2020; Sen et al. 2022). Experiments with human primary hepatocytes and human immortalized hepaRG cells in vitro have shown that occupationally relevant concentrations of PFAS decrease the expression of hepatic nuclear factor 4α (HNF4α) and its downstream effector, the cholesterol-7a-hydroxylase (CYP7A1). HNF4α is regarded as a regulator of many liver-specific processes including liver development, lipid metabolism and maintaining hepatocellular differentiation. CYP7A1 is a key enzyme in bile acid synthesis from cholesterol and therefore the decreased expression of HNF4α and CYP7A1 reduce bile acid synthesis and flow, and may contribute to the development of hepatic steatosis (Beggs et al. 2016; Behr et al. 2020). Moreover, in vitro PFAS induce endoplasmic reticulum stress and alter CYP-enzyme activities (e.g., CYP2C19, CYP2D6, CYP3A4, CYP2E1). Most of the tested PFAS have been shown to inhibit CYP-activities in PFAS structure-dependent and CYP-dependent ways (Louisse et al. 2020; Amstutz et al. 2022).

In rats, exposure to PFAS increases liver tumors (Biegel et al. 2001). In rodent livers, PFAS-exposure activates signaling via the peroxisome proliferator-activated receptor alpha (PPARα) and the expression of genes related to cell cycle, lipid metabolism and transport, apoptosis and cell proliferation (Biegel et al. 2001; Elcombe et al. 2012; Rosen et al. 2017). The increased fatty acid oxidation and proliferation trigger the generation of hydrogen peroxide, oxidative stress and epigenetic chances. In prolonged exposures, these are the main changes considered to be responsible for increased cell proliferation and formation of liver adenoma and carcinoma (Biegel et al. 2001; Elcombe et al. 2012; Butenhoff et al. 2012). In addition, long-chain PFASs inhibit gap-junctional intercellular communication in cell culture models in vitro and in rat liver in vivo (Upham et al. 2009). Cell culture experiments have further indicated that the inhibition is dependent on the activation of extracellular signal-regulated kinase (ERK) and phosphatidylcholine specific phospholipase C (Upham et al. 2009). Inhibition or disruption of gap-junctional communication is a proposed mechanism by which tumor cells avoid growth suppressive signals (for reviews, see e.g., Nahta et al. 2015; Mesnil et al. 2018).

Not only long-chain PFAS but also some short-chain PFAS-alternatives have been reported to induce tumorigenic responses in murine and human livers. Several studies indicate that exposures to these fluorochemicals induce hepatocellular hypertrophy, oxidative stress, activate PPARα-signaling and genes involved in cell cycle and carcinogenic pathways (Sheng et al. 2018; Guo et al. 2022; Heintz et al. 2022; Xie et al. 2022; Thompson et al. 2023). In human primary hepatocyte cultures, GenX disturbs lipid metabolism and induce fibro-inflammatory signaling as well as stimulate growth factors and pro-mitotic pathways (Robarts et al. 2022).

There is evidence that the initial effect of PFAS-exposure is activation of PPARα-receptor-mediated pathways in rodent and human livers. However, the PPARα-receptor in human liver is less responsive to proliferative stimulus of PFAS than the receptor in rodents (Elcombe et al. 2012; Rosenmai et al. 2018; Corton et al. 2018; Attema et al. 2022; Heintz et al. 2023). PPARα belongs to the ligand activated nuclear receptors. It acts as a lipid and xenobiotic sensor regulating energy combustion, lipid homeostasis and inflammation. PPARα modulates the activities of mitochondrial, peroxisomal and microsomal fatty acid oxidation. Sustained activation of this receptor has been linked to hepatocellular carcinoma in rodents (Pyper et al. 2010; Corton et al. 2018; Wagner and Wagner 2022). Gene-profiling studies conducted in rodents have shown evidence that also other nuclear receptors such as PPARγ and CAR/PXR can mediate the PFAS-induced adverse metabolic and carcinogenic changes (Rosen et al. 2017; Attema et al. 2022). For instance, PFOA can alter the expression of genes related to fatty acid and xenobiotic metabolism, inflammation and cell cycle progression in both wild type PPARα and PPARα null mice (Rosen et al. 2017). On the other hand, short-chain GenX and the synthetic PPARα-agonist Wy-14,643 alter the genes in wild PPARα mice but not in PPARα null mice indicating specificity of these compounds to PPARα (Attema et al. 2022). This also implicates that various PFAS-congeners may activate this receptor with subsequent different down-stream effects. Although the permanent activation of PPARα can lead to fatty liver in human, the downstream cell proliferation/growth pathways are generally less activated  than in rodent liver after PFAS-exposure (Elcombe et al. 2012; Corton et al. 2018; Heintz et al. 2023).

Only a few epidemiological studies exist on PFAS-exposure and hepatic tumors. A recent nested case–control study (Goodrich et al. 2022) found that high levels of PFOS is associated with a 4,5-fold elevated risk for hepatocellular carcinoma. Altered metabolism of bile acids, branched-chain amino acids and glucose were suggested as mechanisms affected by PFOS-exposure in human liver. This study identified four metabolites (glucose, butyric acid, α-ketoisovaleric acid and 7α-hydroxy-3-oxo-4-cholestenoate), each of which was positively associated with PFOS-exposure and hepatocellular cancer (Goodrich et al. 2022).

### Effects of PFAS in the kidney

Kidney is another major target organ of PFAS-exposures and important for excretion, and reabsorption of PFAS (Lau et al. 2007; Pérez et al. 2013; Louisse et al. 2023). High blood flow and large endothelial surface per organ weight make kidney vulnerably to chemical insults (Vervaet et al. 2017). In humans, following PFAS exposure indications of kidney failure and injury, such as reduction in the glomerular filtration rate, increase of serum uric acid and tumors have been reported (Shankar et al. 2011; Stanifer et al. 2018; Lu et al. 2019; Shearer et al. 2021). Occupational and large community-based studies have also reported an association between high serum PFAS-levels and a risk of kidney and testicular tumors (Table 1; Barry et al. 2013; Steenland et al. 2020; Bartell and Vieira 2021; Li et al. 2022a; Purdue et al. 2023). The relationship between PFOA, and kidney and testicular tumors have been reported most likely to be causal (Bartell and Vieira 2021).

Experimental in vitro and rodent studies have shown that PFAS induce toxicity and changes in renal histology, metabolism and functions. These include disturbances in lipid and energy metabolism, oxidative stress, increased apoptosis and tubular epithelial hypertrophy (Zhang et al. 2011; Chou et al. 2017; Wen et al. 2021; Lee et al. 2022). PFOS was shown to dose-dependently alter the expression of several genes related to epigenetic pathways in mouse kidney. PFOS up-regulated mRNA of several transcription factors (e.g., Ppara, Ppard, Nef2l2, Hes1) and histone demethylases (e.g., Kdm1a, Kdm4c). In addition, PFOS decreased global DNA-methylation and down-regulated Pparg and Smarca2 (Wen et al. 2022). Epigenetic alterations have also been reported in murine kidney by treatment with PFOA (Rashid et al. 2020). Similar to liver, many adverse effects in the kidney are linked to deregulation of signaling via nuclear receptors, particularly via PPAR-receptors and their down-stream pathways. Use of specific antagonists to PPARα leads to cell cycle arrest (G0/G1) and apoptosis in the human renal epithelial cell line Caki-1 (Aboud et al. 2015). Studies in rat tubular epithelial cells (RTCs) further indicate that PFOS treatment decreases expression of antioxidant enzymes and induce apoptosis through inactivation of the PPARγ-receptor. PFOS treatment was also shown to induce dedifferentiation of tubular epithelial cells with disrupting epithelial cell junctions, leading to increased permeability, partial epithelial-mesenchymal transition and cell migration (Wen et al. 2016; Chou et al. 2017). In addition, mitochondrial disorders e.g., uncoupling of ATP-synthesis and increase of ROS production have been shown to play a critical role in an injury in rat tubular cells in vitro after PFOS-treatment (Wen et al. 2021; Lee et al. 2022).

### Effects of PFASs in the testicle

In rat, PFAS induce tumors in testicular Leydig cells (Biegel et al. 2001; Klaunig et al. 2012). These cells are responsible for synthesizing testosterone that is essential for sexual development in fetal period and for the support of sperm production later (Teerds and Huhtaniemi 2015). PFAS-induced Leydig cell tumors in rodents are strongly linked with hormonal changes principally with the increased ratio of serum estrogen/testosterone levels (Biegel et al. 2001; Klaunig et al. 2012; Zhao et al. 2014). One suggested mechanism to hormonal imbalance is PFAS-caused down-regulation of the expression of testicular steroidogenic enzymes (e.g., Cyp11a1, Cyp17a1, Hsd17b3) as shown in mice (Zhang et al. 2014; Tian et al. 2019; Li et al. 2021). An additional mechanism of PFAS-exposures may be increased expression of the hepatic aromatase enzyme (CYP19A1) that metabolizes testosterone to estrogen (Liu et al. 1996). Hormonal imbalance can increase gonadotropin releasing hormone and LH, which stimulate Leydig cell proliferation. In addition, disturbances in hormonal balance may increase expression of growth factors (e.g., insulin-like growth factors and  transforming growth factor alpha), which may promote cell survival and proliferation of Leydig cells leading to tumor formation (Klaunig et al. 2012; Li et al. 2021). The hormonal imbalance and other effects on Leydig cells have particularly been reported after PFAS-treatment of rodents during prenatal and pubertal periods (Zhao et al. 2014; Li et al. 2021) Abnormalities in sperm quality and lowering of testosterone level by PFAS have been seen in wild type PPARα mice and in the mice containing human PPARα but not in the PPARα-null mice. This implicates that the PPARα-receptor may also be involved in the hormonal imbalance (Li et al. 2011).

Epidemiological studies have reported an association of tumors in the testicle with high serum PFAS levels (Table 1). However, Leydig cell tumors are rare in humans, and testicular tumors refer generally to germ cell tumors, which occur typically in males at 15 to 40-years of age (Klaunig et al. 2012; Skakkebæk et al. 2016). The etiology of germ cell tumors is not fully understood but genetic susceptibility and environmental factors may play an important role. Established risk factors such as low androgen levels, genital malformations, and poor semen quality have been suspected to arise in utero (Skakkebæk et al. 2001, 2016). There is, however, evidence that exposures in adolescence and adulthood to environmental chemicals such as PFAS may increase the risk of testicular tumors (McGlynn and Trabert 2012; Bartell and Vieira 2021; Purdue et al. 2023).

Insufficient androgen levels may also play an important role in the etiology of human testicular cancer (Skakkebaek et al. 2016). Several epidemiological studies have investigated the relationship between sex hormone levels and PFAS-exposure (Lewis et al. 2015; Zhou et al. 2016; Cui et al. 2020; Luo et al. 2021). However, it has been difficult to get conclusive results since PFAS-exposure can affect hormone levels in gender-, age-, and congener-specific manner (Xie et al. 2021). Another mechanism, by which PFAS could disturb endocrine functions, is by interfering with hormone receptors. In vitro experiments provide evidence that PFAS (e.g., PFOA, PFOS, PFHxS, PFNA, and PFDA) inhibit testosterone binding to the androgen receptor (AR). PFOA has also been shown to reduce testosterone-induced translocation of AR to the nucleus, thus eliciting anti-androgenic effect (Kjeldsen and Bonefeld-Jørgensen 2013; Nisio et al. 2019).

### Effects of PFAS in the mammary gland

Breast cancer is the most common cancer in women and exposure to endocrine disrupting chemicals such as PFAS has been regarded as a risk factor in the disease (Gore et al. 2015; Wan et al. 2022). There is some evidence for an association between serum PFAS levels and the risk for breast cancer in non-occupationally exposed women (Table 1). The relationship between high serum PFAS-levels and the risk of breast cancer was reported among Inuit women in Greenland. The consumption of seafood is likely the source for the high levels of PFAS in their serum (Bonefeld-Jørgensen et al. 2011; Wielsøe et al. 2017). Mancini and coworkers (2020) reported a connection between breast cancer and serum PFAS-levels among postmenopausal French women. In that study, serum PFOS-levels were in a positive dose–response relationship with the risk of receptor-positive (ER+ /PR+) breast tumor whereas only low serum concentrations of both PFAS and PFOS were associated with the receptor-negative breast tumor. This study provides evidence that high and low PFAS exposures may affect differently the hormone-dependent tissues (Mancini et al. 2020). In addition, a relationship between serum PFAS-levels and the risk of breast cancer has been reported among women from Asian countries (Table 2; Tsai et al. 2020; Feng et al. 2022; Li et al. 2022a, b; Velarde et al. 2022).

Experimental studies with human breast cell models have revealed that several PFAS (e.g., PFOA, PFOS, PFHxS) have estrogenic properties or they can enhance ER-transactivation and accelerate cell proliferation (Maras et al. 2006; Kjeldsen and Bonefeld-Jørgensen 2013; Sonthithai et al. 2016; Pierozan et al. 2018). In a mixture with two other chemicals (bisphenol-A and methylparaben), environmentally relevant concentration of PFOA increased proliferation of benign breast epithelial cells through disruption of cell cycle, suppression of apoptosis and increasing of ERα-receptor level. The mixture of chemicals perturbed the key pathways more than each individual compound alone implicating that the perturbation would have been missed by evaluating the effects of each single component separately (Dairkee et al. 2018). Low concentrations of a binary mixture (PFOA and PFOS) were also shown to act synergistically and transform breast epithelial cells (MCF-10A) into a malignant phenotype (Pierozan et al. 2023).

Experiments conducted in rodents implicate that PFAS-exposures, particularly during vulnerable developmental periods have adverse effects on endocrine organs and serum sex hormone levels (White et al. 2011; Zhao et al. 2012; Su et al. 2022). Chronic exposure to PFOA alters morphological development of mammary gland and the changes can last over generations (White et al. 2011). Zhao and coworkers (2012) have shown that pre-pubertal PFOA-exposure inhibits growth of the mammary gland in wild type mice but not in PPARα-knockout mice. In addition, exposures to PFOA decreased the levels of several ovarian enzymes (CYP11A1, HSD3β1, HSD17β1), which are involved in biosynthesis of estrogen and progesterone. Recent study by Su and coworkers (2022) also reported that pubertal exposure of rats to a mixture of low-dose PFOA and zearalenone (a mycotoxin) inhibits mammary gland development and increases susceptibility of the animals to DMBA-induced tumors in the mammary gland. This was associated with alterations in estrogen- and Wnt-signaling pathways and was related to the increased expression of growth factors and oncogenes (Su et al. 2022).

Mechanistic studies with pre-neoplastic breast cells (MCF-10A) have further revealed that treatments with PFOS or PFOA alter the levels of cell cycle proteins (e.g., CDK6/4, p21, p53), promote cell proliferation, increase histone modifications, and induce epithelial-mesenchymal transition (EMT) (Pierozan and Karlsson 2018; Pierozan et al. 2018, 2020). During EMT, epithelial cells progressively acquire the morphological and other properties of mesenchymal cells. In this process, cells gain migratory and invasive properties and this leads to metastasis (for a review see Fedele et al. 2022). In the studies of Pierozan and coworkers (2018a, 2018b), the proliferation of MCF-10A cells by PFOA was associated with an activation of the PPARα receptor, while PFOS had no effect on PPARα-signaling as demonstrated by the use of inhibitors of PPARα. Instead, treating the cells with an inhibitor (ICI182,780) of the estrogen receptor α (ERα) was able to partially reduce the stimulating effect of PFOS on cell proliferation (Pierozan et al. 2018). In addition to signaling via ERα and PPARα, crosstalk with other receptors e.g., PXR, and CAR is associated with EMT-pathways in PFAS-exposed breast cells in vitro (Zhao et al. 2012; Pierozan et al. 2022, 2023).

In addition, Pierozan and coworkers (2022) studied the capacity of alternative PFAS-substances to induce proliferation and neoplastic transformation of MCF-10A cells. Only perfluorohexane sulfonate (PFHxS) of the six alternatives (PFHxA, PFO2OA, HFBA, PFBS, GenX, and PFHxS) induced cell migration and invasion and activated the PPARα- and CAR-receptors. PFHxS altered also the expression of proteins that regulate cell cycle and caused histone modifications. However, higher concentrations of PFHxS than PFOA or PFOS were required to induce these effects (Pierozan et al. 2022).

## Summary and conclusions

It is evident from both rodent and human studies that PFAS induce cancer-related molecular changes in several organs. The effects of exposures are complex and depend on many factors such as dose and the chain length of PFAS, as well as species, age and sex of the exposed individuals. In addition, effects of different PFAS-congeners may vary. The main adverse pathways include disturbance of signaling via nuclear receptors, disruption of lipid metabolism and endocrine balance and induction of oxidative stress and epigenetic changes. Signaling via central metabolic regulators such as PPARα, and γ, CAR, PXR, ER and AR are directly or indirectly altered following PFAS-exposure.

Molecular changes in the main PFAS-target organs, liver and kidney, resemble each other in many respects, but vary from the effects in the hormone-dependent organs testicle and breast. Disturbing of signaling via the PPARα-receptor and the expression of its down-stream genes leads to reprogramming of metabolism, particularly lipid and bile acid metabolism. These are the most important initial changes in the liver. In rodents, the changes lead further to proliferation and tumors, whereas in humans the changes can lead to the kind of lipid accumulation that is present in non-alcoholic fatty liver, but proliferation and tumors are not common. In the kidney, PFAS evoke toxicity and change renal functions that include deregulation of signaling via PPAR-receptors, metabolic changes, oxidative stress and epigenetic alterations.

PFAS are also endocrine disruptors modulating hormonal functions in testicle and mammary gland. PFAS-induced insufficient androgen levels and/or anti-androgenic effects are important causes leading to proliferation and tumors in rodent testicular Leydig cells. There is some data suggesting that similar molecular changes are associated with human testicular cancer. However, the precise mechanisms are not known. Several PFAS have estrogenic properties and they can enhance estrogenic responses and increase susceptibility to other carcinogens in the mammary gland. The mechanisms include signaling via ER and PPARα-receptors as well as crosstalk with other nuclear receptors, increase in cell proliferation and suppression of apoptosis. Timing of the exposure is important and it seems that exposure to PFAS during prenatal and postnatal periods may disrupt mammary gland development. The effects may last across different generations. In addition, PFAS-exposure inhibits intercellular communication through gap-junctions and induces EMT. These alterations promote the development of a malignant cell phenotype enabling migration and metastasis.

PFAS-induced tumorigenic changes are significant from public health perspective because most individuals are exposed to these persistent chemicals in everyday life. Moreover, most of our knowledge on the involvement of PFAS in tumors is from studies with a few long-chain PFAS compounds. Other PFAS-congeners and especially PFAS mixtures need more research. Current results raise particularly concerns about potential consequences of PFAS-exposure in endocrine system during developmental period, which also needs more research.

## Data Availability

Data sharing not applicable to this article as no dataset were generated or analyzed during the current study.
